# Zero Suicide Campaign: Establishing a National Suicide Prevention Hotline in Conflict-Affected Myanmar

**DOI:** 10.1192/bjo.2026.11447

**Published:** 2026-06-30

**Authors:** Maung Oakarr, Moon Monica, T. Tina, W. Winnie, H. White

**Affiliations:** Myanmar Board of Psychiatry, Rangoon, Myanmar

## Abstract

**Aims:**

Following the military coup in February 2021, Myanmar has experienced widespread human rights violations, insecurity, economic hardship, and disruption of essential services, resulting in a substantial deterioration in population mental health. Media-reported suicides have increased, alongside rising mental health presentations within TeleKyanmar, a nationwide telemedicine service operating under the Ministry of Health, National Unity Government (MOH-NUG). In response, TeleKyanmar launched the Zero Suicide Campaign (ZSC), Myanmar’s first national suicide prevention hotline, in September 2023.

The primary objective of ZSC is to reduce suicide risk through timely crisis intervention. Secondary objectives include narrowing the mental health treatment gap and facilitating rapid referral to appropriate mental health services for individuals at risk.

**Methods:**

The preparation phase (December 2022–August 2023) involved multi-stakeholder consultation with mental health and MHPSS experts, followed by three rounds of virtual suicide prevention training (April–June 2023). A total of 168 Civil Disobedience Movement (CDM) professionals completed foundational training, from whom 50 were selected for advanced applied training. The applied programme comprised 24 hours of structured teaching and supervised skills practice, focusing on suicide risk assessment, safety planning, empathic listening, crisis hotline ethics, and operational challenges.

The hotline was launched on World Suicide Prevention Day (10 September 2023) using the Telegram platform. It operates seven days a week across two daily shifts, with three parallel lines. Trained volunteers provide crisis support, supported by live and bi-weekly psychiatric supervision. Standard Operating Procedures guide case management and referrals. Suicide risk is assessed using the Columbia Suicide Severity Rating Scale (C-SSRS) and safety planning is conducted using the Stanley–Brown Safety Plan.

**Results:**

Currently, 41 trained volunteers provide the service. Individuals assessed as medium or high suicide risk are referred to TeleKyanmar mental health clinics within 24–72 hours, while those at lower risk are signposted to community-based online counselling services. The hotline enables anonymous, stigma-reduced access and functions as an effective gateway into structured mental healthcare.

**Conclusion:**

The Zero Suicide Campaign represents a scalable, low-cost public health intervention in a conflict setting. It provides immediate crisis support, reduces barriers to care, and strengthens suicide prevention pathways within a disrupted health system. The model demonstrates how telemedicine-linked crisis services can mitigate suicide risk in humanitarian emergencies.

Acknowledgement: We acknowledge all Civil Disobedience Movement mental health professionals and members of the Myanmar Psychiatrist Diaspora whose voluntary commitment made the Zero Suicide Campaign possible.

